# Effect of lockdown on activities of daily living in the built environment and wellbeing

**DOI:** 10.14324/111.444/ucloe.000017

**Published:** 2021-04-21

**Authors:** Sudhir Kumar Pasala, Lakshmi Gumpeny, Madhu Kosuri, Snehalatha Tippana, Gumpeny R. Sridhar

**Affiliations:** 1 Department of Architecture, Andhra University College of Engineering (Autonomous), Visakhapatnam, India; 2 Department of General Medicine, Gayatri Vidya Parishad Institute of Healthcare & Medical Technology, Visakhapatnam, India; 3 Department of Psychology and Parapsychology, Andhra University / Wellness Hub, Visakhapatnam, India; 4 Kendriya Vidyalaya Sangathan, Vizianagaram, India; 5 Department of Endocrinology, Endocrine and Diabetes Centre, Visakhapatnam, India

**Keywords:** work from home, diet, sleep, stress, entertainment, television viewing, built environment

## Abstract

In an effort to arrest the spread of coronavirus (COVID-19) infection, a nationwide lockdown was declared in India in March 2020. To assess how personal built environments affected the citizens in the first few weeks, an explorative online survey was conducted, eliciting responses about work habits before the lockdown, psychological wellbeing, time spent in various activities, characteristics of those who worked from home, and food and sleep patterns. We received 121 (76 male and 45 female) responses with an average age of 35.5 years [max: 70 years, min: 18 years, standard deviation (SD): 12.9 years]. The major difference caused by the lockdown was a reduction in the time taken and distance travelled of the commute to workplaces, which was an average of 30 minutes and 9.5 km, respectively. In terms of diet, subjects who were vegetarian did not experience any difference, unlike those who were non-vegetarians (*p* < 0.05). The results show an association of the dependent variable of ‘feeling in general’ with predictor variables of ‘energy, pep, vitality’ and ‘feel healthy to work’ during the pandemic, whereas the predictor variables of ‘energy, pep, vitality’, ‘happy and satisfied personal life’, ‘feel healthy to work’ show an association with the dependent variable of ‘feeling in general’ before the lockdown with a significance of *p* < 0.02 and R^2^ = 0.51 and R^2^ = 0.60, respectively. Among those who worked from home in constrained environments, people found spaces and seemed to adapt reasonably well to the built environment with employees showing a preference for working from bedrooms and students for working from ‘sit-out’ (outside) spaces (*p* < 0.05). There was no change in the quality or quantity of sleep during the lockdown. This study in the early weeks of the lockdown documents the way in which individuals lived through it in terms of the built environment at home.

## Introduction

The coronavirus (COVID-19) epidemic, identified at the beginning of 2020 has the ability to spread by droplet transmission. In the initial phase of the pandemic, when the study was carried out, the only measures to reduce the transmission of the virus consisted of physical distancing, frequent washing of hands with soap and water, and avoiding touching one’s face. These are still the core preventive measures even after vaccines and potential medicines became available to treat the infection.

Although the physical measures are simple to itemise, they are difficult to implement. In an attempt to prevent the community spread of infection, India imposed a lockdown, beginning on 22 March 2020. Depending on the situation, the lockdown has been modified over time.

Although the lockdown was for the common good, the uncertainty about the disease coupled with nationwide lockdown led to a stressful situation. It is understandable that apprehension and anxiety could result from loneliness due to social isolation, a fear of being infected, the resultant economic impact and uncertainty about the future course [1]. A report that compared psychological distress and loneliness in 2018 and in April 2020 showed that the prevalence of serious psychological distress increased three-fold in April 2020 [1].

Following the outbreak of COVID-19, a number of studies were published on knowledge, attitude and practices (KAP) about the conditions across the globe, including different parts of India [2–4].

The built environment refers to ‘environments that are modified by humans, including homes, schools, workplaces, highways, urban sprawl, accessibility to amenities, leisure and pollution’ [5]. It is conceivable that the response to the pandemic and measures to slow its spread can be modified by the built environment. To the best of our knowledge, there have not been any studies evaluating the effect of the built environment on daily living and psychological stress during the lockdown. A report from Brazil studied the spatial correlation between the incidence of COVID-19 and human development [6]. Doshi et al. reported that fear about COVID-19 was low due to a lack of knowledge, although being a woman, having a lower educational status and being a health care worker were associated with higher fear levels [7]. In situations such as these, in-person interviews are neither feasible nor desirable. Earlier studies have shown that social media platforms can be employed to recruit as well as to communicate about COVID in both developed and developing countries [8,9]. Therefore, we conducted an online survey to assess the effect on living habits, attitudes, and other aspects influenced by the built environment during the early weeks of the lockdown.

The twin aims of the study were to evaluate how activities of daily living (ADL) have had a bearing on wellbeing during the lockdown and how spaces at home have supported ADL during the ‘stay home stay safe’ strategy. The research questions we attempt to address are: (A) Is there a perceptual change in wellbeing during lockdown to that of before lockdown? (B) As a health concern, are there any changes in food habits and rest/sleep? (C) How do people accomplish their responsibilities of work/study?

## Methods

The second phase of lockdown, which began on 15 April 2020 and lasted until 3 May 2020, had stringent restrictions of the instruction to ‘stay home’, with 3–4 hours of relaxed restrictions in the morning to enable citizens to acquire essential commodities. A structured questionnaire was developed covering different sections in sequence, namely demographics, food intake, ADL, the built environment (specifically homes), leisure and entertainment, and health and wellbeing.

**Figure 1:**
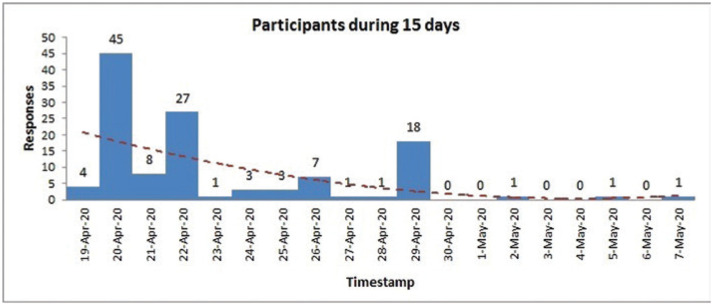
Duration of online survey.

This self-reported questionnaire survey designed in Google forms (available in the Appendix) was administered online from 19 April 2020 to 7 May 2020, that is, during and four days beyond the end of the second phase of lockdown (Fig. 1). The online questionnaire was circulated to the contacts of the authors using online social media.

The section on Demographics has data pertaining to Age, Gender, Height, Weight, Marital Status, Education and Employment. The Food Intake section is related to information on changes of intake of food during principal meals and any change in intake of vegetarian and non-vegetarian food items. Information on ADL covered day-to-day tasks. Questions in the Built Environment section related to where their residence is located (area, floor level), type of house (rented/owned, individual/apartment. etc.), what spaces does a respondent have, and where they spent most of their time during lockdown. As watching television (TV) and spending time with family at home were common leisure and entertainment activities, questions were included on the preferences on which TV channels were watched, such as movies, sports, education, spiritual, serials/drama, music, environment and news. Lastly, the section on Health and Wellbeing relate to whether they were taking any medication along with six questions on wellbeing (feeling in general; energy, pep or vitality; feel any tension; happy, satisfied or pleased with personal life; feel healthy enough; and concerned or worried about health and wellbeing). Following the objectives of the study, the wellbeing of the subjects were assessed for ADL and how spaces at home supported them using a linear regression.

### Statistical analysis

Of the 121 responses received, there was a considerable demographic representation of age, gender, food habits and profession (Table 1). For a mean age of 35.5 years (max: 70.0; min: 18.0; standard deviation [SD]: 12.9) the mean body mass index (BMI), a physiological parameter was 26.3 (max: 49.9; min: 15.7; SD: 4.6).

**Table 1.** Details of responses by gender, food habits and profession.

	Gender		Food habits		Profession		
	Male	Female	Vegetarian	Non-vegetarian	Employee	Student	Home based
Percentage	63%	37%	27%	73%	70%	22%	8%
Number	76	45	33	88	85	27	9

The study was conducted in the Andhra Pradesh region of India which constitutes 10.37% of the COVID-19 confirmed cases of the total confirmed cases in the country. The population of the state of Andhra Pradesh is 52 million constituting 0.04% of the 135 million population of India. The state ranks second in the total number of confirmed cases of COVID-19 in India [10]. With the prevailing limitations for conducting a physical survey and limited access to online survey methods due to India being a developing country, there were 121 respondents with a composition of mean age of 35.5 years (max: 70 years–min: 18 years), 63% were male and 37% were female, 92% were work related and 08% were home-based and 73% were non-vegetarians and 27% were vegetarians. Work related means the major activities or the profession of an individual involves them being away from home and includes employment and education. The margin of error is 7% for the sample size and the population of the study with a 90% confidence interval (CI) and thus the results differ within 7 percentage points from the real population value 90% of the time. Linear or multiple regression analysis was employed to evaluate the relationship of the dependent variables with predictor variables. Independent variables which have significance of *p* < 0.05 with coefficients that represent an association with the dependent variables are discussed. Smaller values of R^2^ may not necessarily be insignificant, although caution must be exercised in interpretation without being combined with other statistical methods. However, based on the knowledge of the subject area in studies of human behaviour, which are difficult to predict, a high value of R^2^ has been described as being ‘almost impossible’ [11]. Given this caveat, the results at least show a trend that can be further studied. The variables considered throw light on aspects that could be taken into account to find ways to live with situations like the COVID-19 pandemic. Statistical analysis using Excel was carried out for the parameters of demographics, food intake, ADL, the built environment, leisure and entertainment, and health and wellbeing.

## Results

### Food habits

Questions on whether there was any change in food intake in main courses, viz. breakfast, lunch, evening snacks and dinner were considered. The respondents were asked whether there had been any increase or decrease of food intake or if food intake had remained the same during and before the lockdown. Also information was taken on what type and quantity of vegetarian and non-vegetarian food was consumed.

**Figure 2:**
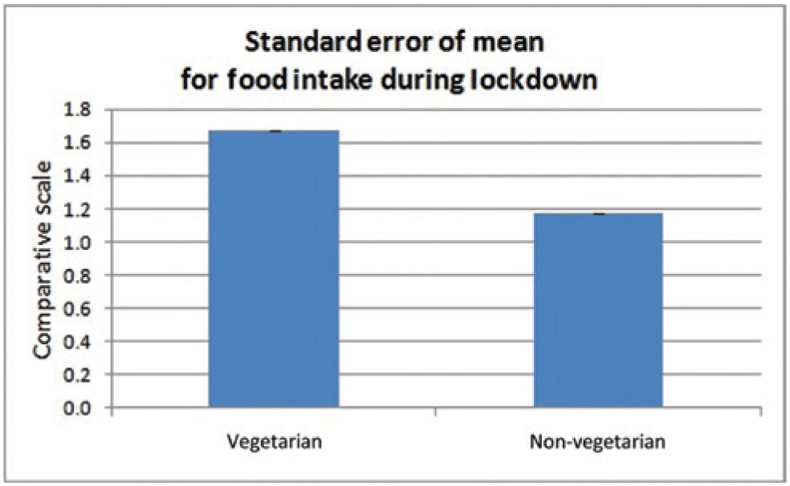
Food intake during lockdown.

The cumulative quantity in terms of ‘increase’, ‘decrease’ or ‘remained the same’ for various food items in vegetarian and non-vegetarian groups was divided into a number of items for the respective groups to normalise. Items such as chicken, mutton, fish, etc. were considered for non-vegetarians and various types of leafy vegetables, tubers, vegetables, etc. were considered for vegetarians. Figure 2 shows the main course and type and quantity of food items by these respective groups and this should ideally be the same value. The t-test for vegetarian and non-vegetarian groups show significant differences (*p* < 0.05) for food intake during the lockdown. Furthermore, the standard error of mean for the two groups of vegetarian and non-vegetarian food intake during lockdown shows a reduction in intake of non-vegetarian items (Fig. 2).

### Health and wellbeing

Self-reported questions on perception of wellbeing parameters include ‘energy, pep, vitality’, ‘happy and satisfied personal life’, ‘feel healthy to work’, ‘generally tense’ and ‘worried about health’. However, a question on ‘feeling in general’ was asked which included overall perception of health. A regression analysis of different predictor wellbeing parameters that contribute to ‘feeling in general’ was analysed for both before and during lockdown. The results of 121 subjects show an association of the dependent variable of ‘feeling in general’ with a significance of *p* < 0.02, R^2^ = 0.51 to predictor variables of ‘energy, pep, vitality’ and ‘feel healthy to work’ with a positive coefficient of 0.305 and 0.374, respectively, during the pandemic situation.

Whereas the predictor variables of ‘energy, pep, vitality’, ‘happy and satisfied personal life’, ‘feel healthy to work’ show significant relation, *p* < 0.02, with the dependent variable of ‘feeling in general’ observed before lockdown with R^2^ = 0.60, indicating a greater reliability with positive coefficients of predictor variables of 0.501, 0.193, 0.207, respectively. It is expected that ‘energy, pep or vitality’, ‘happy and satisfied personal life’ and ‘feel healthy to work’ are associated with ‘feeling in general’ before the lockdown in the absence or nonexistence of the disease. With the emergence of the epidemic and the promulgation of stay at home measures the association of ‘feeling in general’ is maintained in ‘energy, pep or vitality’ and ‘feel healthy to work’, albeit, with a mean change in coefficients of the predictor variables. While there was reduction in coefficient of ‘energy, pep or vitality’ compared with feelings before lockdown, the increase in the coefficient for ‘feel healthy to work’ during lockdown could be attributed to improved safe conditions of work from home (WfH). It is also strengthened by the fact that an association of a ‘happy and satisfied personal life’ with ‘feeling in general’ was noticed before lockdown (Table 2).

**Table 2.** Regression results.

Dependent variable	Predictor variable	Coefficients	Standard error	t Stat	*p*-Value
Feeling in general	During lockdown				
Sample size = 121	Energy, pep or vitality	0.305	0.122	2.504	0.014**
R^2^ = 0.509	Happy, satisfied personal life	0.149	0.096	1.556	0.123
Significance, F = 2.21E-16	Feel healthy to work	0.374	0.098	3.802	0.000**
Intercept = 1.704	Generally tense	−0.115	0.072	−1.586	0.115
	Worried about health	−0.124	0.073	−1.699	0.092
Feeling in general	Before lockdown				
Sample size = 121	Energy, pep or vitality	0.501	0.088	5.687	0.000**
R^2^ = 0.601	Happy, satisfied personal life	0.193	0.071	2.739	0.007**
Significance, F = 1.89E-21	Feel healthy to work	0.207	0.083	2.484	0.014**
Intercept = 1.189	Generally tense	−0.066	0.051	−1.285	0.202
	Worried about health	−0.08	0.056	−1.441	0.152
Feeling in general	Watching TV (news channels)				
Sample size = 121	News updates on COVID-19 cases	0.269	0.126	2.13	0.035**
R^2^ = 0.189	News updates on COVID-19 health precautions	−0.173	0.14	−1.234	0.22
Significance, F = 0.00002	General new updates	0.311	0.113	2.74	0.007**
Intercept = 3.383					
Happy, satisfied personal life	Watching TV (leisure channels)				
Sample size = 121	Movies	0.155	0.08	1.938	0.055
R^2^ = 0.145	Music	0.055	0.08	0.689	0.492
Significance, F = 0.001	Spirituality	0.201	0.077	2.61	0.010**
Intercept = 4.069					
Feel healthy to work	Mode of transport				
Sample size = 121	Public transport (bus/metro, etc.)	−0.19	0.114	−1.665	0.099
R^2^ = 0.119	Para transport (autorickshaw)	0.115	0.136	0.85	0.397
Significance, F = 0.023	Company vehicle	0.066	0.076	0.871	0.385
Intercept = 4.112	Personal car	0.161	0.067	2.395	0.018**
	Personal 2-wheeler	0.084	0.068	1.236	0.219
	Shared transport (friend’s vehicle)	0.014	0.083	0.174	0.862
WfH	Employees living at individual houses irrespective of ownership				
Sample size = 22	Bedroom	3.253	1.355	2.401	0.029**
R^2^ = 0.526	Balcony/sit-out/utility	−1.631	0.815	−2.001	0.063
Significance, F = 0.024	Front/back yard	−1.932	1.379	−1.401	0.18
Intercept = 10.698	Toilet	−1.752	1.131	−1.55	0.141
	Other rooms	−1.746	0.958	−1.823	0.087
WfH	Students staying at own houses				
Sample size = 21	Bedroom	1.54	1.041	1.48	0.16
R^2^ = 0.481	Balcony/sit-out/utility	1.727	0.803	2.152	0.048**
Significance, F = 0.057	Front/back yard	−2.164	1.212	−1.785	0.095
Intercept = 7.882	Toilet	−2.488	1.03	−2.415	0.029**
	Other rooms	−0.717	0.799	−0.898	0.384

**Indicates significance at 95% CI.

CI, confidence interval; WfH, work from home.

### Watching television

There is a significant positive relation with 95% CI and R^2^ = 0.18 to ‘feeling in general’ for watching the news channels for ‘news updates on COVID-19 cases’ with (*p* < 0.05) and ‘general news updates’ with (*p* < 0.01) and increasing trends of 0.269 and 0.311 coefficients, respectively (Table 2). There is a significant relation to a ‘happy and satisfied personal life’ with (*p* < 0.01) at 95% CI and R^2^ = 0.14 for channels related to ‘spirituality’ with an increasing trend of 0.201 coefficient with the dependent variable.

### Mode of transport

The expectations of people when the lockdown ends shows that the mode of transport of using one’s own car has a positive coefficient of 0.161 with ‘feel healthy to work’ with a significance of (*p* < 0.02) with R^2^ = 0.12 (Table 2).

### Built environment and work from home

The average distance of 9.5 km and an average 30-minute travel time by respondents to an office/educational institute saved them time and energy during lockdown that could instead be contributed to WfH [12]. We assessed the relationship to home with WfH in two different aspects. One being the ownership of the house (whether rented, owned or quarters provided by the employer) and the other was the typology of the building (individual house, apartment/group housing and row housing). Group housing is a type of housing consisting of four to 12 tenements in a building whereas an apartment block has more than 12 tenements in a building. In the 121 samples surveyed, there are three categories of ownership of which 62% of them owned their own residence, 37.2% lived in rented houses and 0.8% lived in quarters provided by the employer. As regards the typology of building, 52.9% were apartment/group housing, 40.5% were individual/independent houses and 6.6% were row housing. However, quarters provided by the employer in ownership category and row housing in typology of the building were not considered due to small sample size. Also there were 22 home-based (10 homemakers/retired persons and 12 office/businesses operating from home) that were not considered.

**Figure 3:**
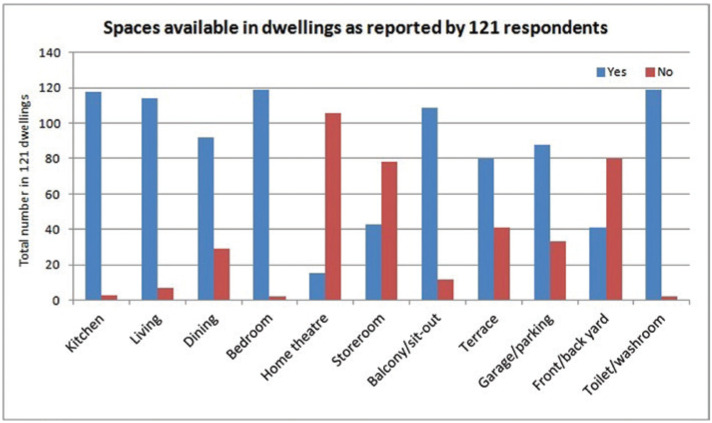
Spaces in dwellings.

The generally available spaces in residential buildings in India are a kitchen, a living room, a dining room, a balcony or sit-out space, and a toilet/washroom. More than 90% of residential buildings have between one and four bedrooms [13]. Spaces such as living and dining rooms are noisy. Often, living and dining spaces where multiple activities take place are interconnected in India. Relatively quieter spaces such as the balcony/sit-out spaces are mostly used as micro-gardens and for relaxation for short durations, while the quietest area is the bedroom. It is important to find a suitable place to work at home. The number of dwellings studied consisting of these spaces are shown in the figure (Yes – available and No – not available) with few having exclusive spaces viz. storage space, home theatre, garage/parking, terrace and back/front yard (Fig. 3).

We assessed the response of the participants on WfH and found that people whose homes are of the group housing/apartment type that constitute 52.9% of those surveyed have no significant relation, which seemingly reflects unfavourable conditions. As mentioned earlier, group housing is a type of housing consisting of four to 12 tenements in a building whereas an apartment block has more than 12 tenements in a building.

Those staying in individual houses (22 respondents) irrespective of the ownership prefer to WfH from their bedroom space (*p* < 0.05 and R^2^ = 0.52) with 95% CI and a positive 3.253 coefficient (Table 2). As for the students, those who stay in their own houses (21 samples) prefer balconies/sit-out spaces when performing their work (*p* < 0.05 and R^2^ = 0.48 with 95% CI) with a positive coefficient of 1.727 with the dependent variable WfH. Interestingly, for the students the association of the toilet/washing area with WfH (*p* < 0.05 and R^2^ = 0.48, 95% CI) and −2.488 coefficient although negative, reflects its importance during work time. It is likely that toilet/washing areas in institutes are used by a greater number of people, specifically in India and during COVID-19 this will be a concern for students. And it is reasonable to anticipate that the availability of a toilet/washing area is negatively associated with WfH compared to that available at institutes. Moreover, there is a concern for the hygiene required during COVID-19 for toilets/washing areas at institutes compared with those at home.

**Figure 4:**
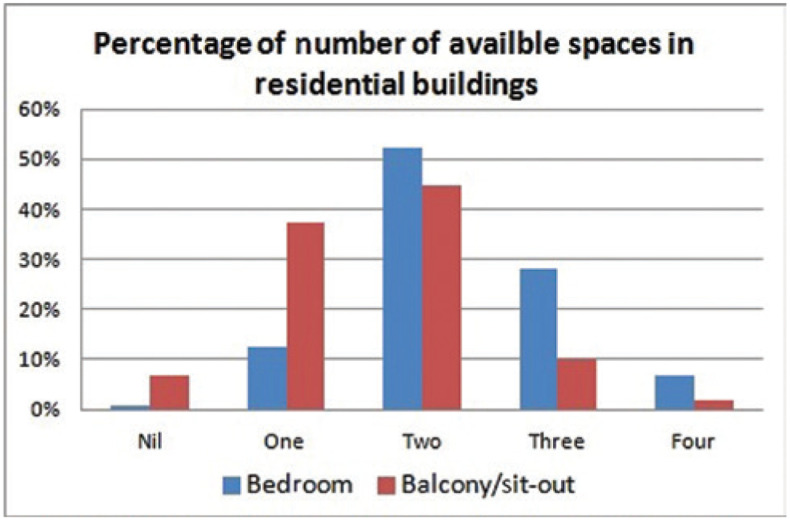
Percentage of the number of bedrooms and balcony/sit-out spaces present in dwellings.

Therefore, the general living conditions in Indian homes can broadly be categorised into active and passive zones. Activities related to watching TV, family interactions and daily household chores are performed in the active zones that include the living room, dining room and kitchen and these are often noisy. Hence, with no other choice left, the possibility of WfH most likely happens in bedrooms and sit-out spaces that are relatively calm and are sufficient in number considering the average size of four members in a family (Fig. 4). However, the design of spaces that could accommodate the requirements of formal and calm environments for WfH is important during situations of ‘stay home stay safe’.

## Discussion

We report an exploratory review of how the built environment was impacted by the world’s biggest lockdown following the COVID-19 pandemic; rather than taking a narrow technical view of architecture per se in terms of construction, transmissibility and other micro-environmental factors, we considered the different ways in which people responded to working from home in their immediate built environment.

The average distance of 9.5 km and average 30-minute travel time of the respondents to the office/educational institute have actually saved them time and energy that could be contributed to WfH. Essentially, we observed that the major difference entailed by the lockdown was a reduction of time and travel to go to their workplace. In terms of food intake, those who ate only vegetarian food did not experience any change, unlike those who were non-vegetarians, who reduced their intake of meat. This was necessitated both by the cost as well as an (unfounded) fear of transmission of infection through meat. There is a fear amongst people that wet marketplaces are a potential threat of transmission of diseases such as COVID-19; as such there is a need for a transformational change in the way they function. Those involved in creating the built environment could rethink strategies for the location and design of wet marketplaces to enhance how they function and offer safe and healthy places for people to live.

Watching TV at home was a common pastime to fill the time available during the lockdown. Forced social isolation did not alter the channels watched (movies, sports, educational, spiritual, soap operas, music, environmental or the news). Watching the general news and COVID-19 updates seems to have positive affects along with watching channels devoted to spirituality. However, there is a need to consider how the design of homes, building construction and materials could support multiple activities in the home and help with WfH. Acoustics and the Internet of Things amongst others could become integral design considerations for those involved in designing the built environment.

The unprecedented lockdown led to families staying at home, and accomplishing all their usual activities in an environment for which it was not originally designed, viz. employment work, studies, entertainment and leisure all at once by all the family members. Among those who worked from home, most preferred to work from their bedroom. Students preferred to study outside the house, on balconies or in sit-outs.

Sleep is often compromised in the modern world, where people are accused of ‘gorging themselves with food and starving themselves of sleep’ [14]. The pandemic was a situation where there was ample time available for sleep/rest, without the distractions of work or the forced circadian disruptions of shift work. However there does not seem to have been any adverse effect, but the small sample sizes make it difficult to reach valid conclusions. However, factors including fear of being infected and economic uncertainty could have played a major role. It was a period of forced isolation, not a volitional vacation; in addition the period of study could have been too short for any changes to be perceived.

Following the recognition of the COVID-19 pandemic, attention has focused on built environment trends to lower the risk of transmission in the design of buildings [15], as well as other tactile surfaces such as doorknobs, switches, toilet handles and faucet knobs [16]. More broad-based concerns about the construction of smart cities which can deal with future pandemics consisted of the popularisation of health science, improving emergency health systems and continuing multi-industry coordination mechanisms, to deal with pandemics [17].

The concept and application of the built environment owes its origin to epidemics and pandemics in the past: bubonic plague in the 14th century, yellow fever in the 18th century, and cholera and smallpox in the 19th century all resulted in innovations such as broad boulevards, sewer systems, plumbing and urban sprawl [18].

Besides healthy workplaces, telecommuting and online accessibility of various services including telemedicine, distance learning, online shopping and online entertainment are bound to evolve. Houses are not just physical structures, they are part of a broader social sphere; pandemics disturb the structures and routines that are closely inter-related, which is an interesting macro feature to consider [19]. Some of the potential ways COVID-19 will impact the built environment consist of a shift away from large city offices, a reduced reliance on cars for transport to jobs and the development of new forms of public spaces [20].

Ultimately these must lead to a rethinking of design, operations, behaviour and maintenance to ensure that first the workplace and thereafter the economy are less susceptible to disruptions caused by disease [21].

To convert the crisis into an opportunity, one must plan to respond to such unexpected events by recalibrating transport facilities, improving spatial distancing in workplaces, as well as redesigning the environment by fusing blue and green infrastructures [22,23].

## Conclusion

The unprecedented lockdown due to the COVID-19 pandemic has greatly impacted the behaviour of families staying at home and accomplishing all their usual activities in an environment for which it was not originally designed. The ‘stay home stay safe’ strategy contributed to wellbeing factors of general health, happiness and vitality while alleviating feelings of worry about health and feeling tense.

There seems to have been some influence of ‘energy, pep or vitality’ and ‘feel healthy to work’. The coefficient, ‘energy, pep or vitality’ seems to have had an increased effect before the lockdown when compared to during the lockdown and for ‘feel healthy to work’ it seems to have improved during lockdown as a result of being in a safe WfH situation.

However, the predictor variable of ‘happy, satisfied personal life’ was prevalent before the lockdown.

In terms of food intake, those who ate only vegetarian food did not experience any change, unlike those who were non-vegetarians, who reduced their intake of meat. This was necessitated both by the cost as well as an (unfounded) fear of transmission through meat. The fear amongst people that wet marketplaces are a potential threat of transmission of diseases such as COVID-19, highlighted that there is a need for transformational change in the way they function. The professionals of the built environment could rethink strategies for the location and design of wet marketplaces to enhance the way they function and offer safe and healthy places. With ample time to rest there does not seem to have been any effect on sleep prior to the lockdown, that is, during normal days and during lockdown.

The average distance of 9.5 km and 30-minute time travel to the office/educational institute have actually saved time and energy to contribute to WfH. The relationship of home with WfH by ownership and typology of the building show that those staying in individual houses irrespective of the ownership prefer WfH from their bedroom space, whereas for the students, those who stay at their own houses prefer balconies/sit-out spaces to perform their activities. However, the general living conditions in Indian homes with family interactions mostly occurring in the living and dining rooms and in kitchens that are often noisy, shows how the design of spaces that could accommodate the requirements of formal and calm environments for WfH is important during situations of ‘stay home stay safe’. There is a need to consider how the design of homes, building construction and materials could support the multiple activities of the home and work. Acoustics and the Internet of Things among others could become integral design considerations for those involved in designing the built environment.

Some of the potential ways COVID-19 will impact the built environment consist of a shift away from large city offices, modes of transport and the development of new forms of public spaces. More broad-based concerns about the construction of smart cities which can deal with future pandemics consisted of the popularisation of health science, improving emergency health systems and keeping in place multi-industry coordination mechanisms, to deal with pandemics. Besides healthy workplaces, telecommuting and online accessibility of various services including telemedicine, distance learning, online shopping and online entertainment are bound to evolve.

### Limitation of the study

Our exploratory study has limitations in having a small sample of subjects along with inherent biases in the recruitment of subjects who had access to the internet, were conversant in English and agreed to participate in the study. The method of the online questionnaire circulated to the contacts of the authors on social media was adopted from studies under similar situations [8]. Nevertheless, it confirms the principles the built environment have on wellbeing and health [24] and hopefully provides an impetus for developments based on sound biopsychosocial concepts.

## Data Availability

The datasets generated during and/or analysed during the current study are available from the corresponding author on reasonable request.

## Appendix

Online link to Questionnaire: https://forms.gle/1dBc9s8NWK4WrZQe6
